# F139. INCLINATION OF STIGMA TOWARD SCHIZOPHRENIA, ATTENUATED PSYCHOSIS, PSYCHOTIC-LIKE EXPERIENCES AND DEPRESSION AMONG DIFFERENT SUBPOPULATIONS IN TAIWAN

**DOI:** 10.1093/schbul/sby017.670

**Published:** 2018-04-01

**Authors:** Chen-Chung Liu, Yen-Chin Wang

**Affiliations:** 1 National Taiwan University Hospital and College of Medicine, National Taiwan University; 2 National Taiwan University Hospital

## Abstract

**Background:**

Stigma toward mental illness may lead to delayed detection, impaired treatment adherence, and poorer recovery. The fear of being labeled as at risk of psychosis can be barriers to early identification of putative prodromal subjects. Compounded by an intriguing feature, psychotic-like experience with no apparent impact on functioning, we wonder if people will generalize their prejudice and discrimination towards schizophrenia to subjects with subthreshold psychotic symptoms.

**Methods:**

A cross-sectional survey using a structuralized questionnaire modified from a previous study conducted by the Hong Kong University Early Psychosis research team was employed. Participants were recruited from various channels; including laypersons in general population invited after their attending talks about mental health topics, patients with mental illness and their key caregivers of 2 hospitals, and mental health professionals. The key component of this questionnaire is comprised by 4 case vignettes describing the symptoms and disabilities of patients with attenuated psychosis syndrome (APS), schizophrenia, depression, and psychotic-like experiences (PLE), respectively; followed by 2 sets of questions using 4-point Likert scale with 19 and 21 items for each set to measure social distance as a proxy for discrimination and prejudices. Basic demographic information, including age, gender, education level, current occupation, and previous contact with persons with mental illness were also collected.

**Results:**

A total of 354 subjects completed this survey, including 239 lay publics, 32 psychiatric patients, 29 patient’s main caregivers, and 54 mental health professionals. Stigmas, especially prejudice, toward PLE are significantly higher in the patient group compared to the other 3 groups. Prejudice, but not discrimination, toward depression is significantly lower in professionals group. Stigmas toward schizophrenia and APS are in general not significantly different among groups, although the general public showed marginally higher scores in discrimination compared to the patient group. In each individual group, the patterns of attitude reflecting discrimination and prejudice are almost identical; that is, highest toward schizophrenia, followed by APS and depression (almost equivalent to each other), and lowest toward PLE, except the patient group which failed to reveal significant differences in ratings of 4 clinical case vignettes.

**Discussion:**

APS seems to be a clinical entity too new to be judged. The majority of participants, except the patient group, reported a similar gradient of stigma toward different clinical severities. Such a result is consistent with previous studies that APS shared similar level of stigma with depression, not as high stigma as towards schizophrenia, but higher than PLE. Interestingly, patients with psychosis might have assimilated PLE to symptoms of schizophrenia based on their personal experiences, so they might have overrated the severity of subjects with PLE.

